# Evaluation of Thermophysical and Mechanical Properties of Sandstone Due to High-Temperature

**DOI:** 10.3390/ma15238692

**Published:** 2022-12-06

**Authors:** Zhen Dong, Yanpeng Chen, Xinggang Wang, Lingfeng Kong, Lianguo Wang, Xinning Li, Fenjin Sun, Ke Ding, Hanqi Wu, Shanshan Chen, Mengyuan Zhang

**Affiliations:** 1 Research Institute of Petroleum Exploration & Development, PetroChina, Beijing 100083, China; 2 Research Institute of Petroleum Exploration & Development, PetroChina Tuha Oilfield Company, Hami 839009, China; 3 Petrochina Planning Department, Beijing 100024, China; 4 State Key Laboratory for Geomechanics and Deep Underground Engineering, China University of Mining and Technology, Xuzhou 221116, China; 5 Petrochina Gas Storage Company, Beijing 100101, China

**Keywords:** underground coal gasification, high-temperature sandstone, thermophysical properties, mechanical properties, microstructure

## Abstract

In this study, thermophysical and mechanical tests were conducted on sandstone samples from room temperature to 1000 °C. Based on the test results, the thermophysical properties (such as specific heat capacity, thermal conductivity, and thermal expansion coefficient) of sandstone after high-temperature treatment and the variations of mechanical properties (including peak strength, peak strain, elastic modulus, and whole stress-strain curve) with temperature were analyzed. Indeed, the deterioration law of sandstone after high-temperature treatment was also explored with the aid of a scanning electron microscope (SEM). The results show that with the increase in temperature, the specific heat capacity and thermal expansion coefficient of sandstone samples after high-temperature treatment increase first and then decrease, while the thermal conductivity gradually decreases. The range from room temperature to 1000 °C witnesses the following changes: As temperature rises, the peak strength of sandstone rises initially and falls subsequently; the elastic modulus drops; the peak strain increases at an accelerated rate. Temperature change has a significant effect on the deterioration rules of sandstone, and the increase in temperature contributes to the transition in the failure mode of sandstone from brittle failure to ductile failure. The experimental study on the thermophysical and mechanical properties of sandstone under the action of high temperature and overburden pressure has a guiding significance for the site selection and safety evaluation of UCG projects.

## 1. Introduction

Underground coal gasification (UCG) is an in-situ fluidized mining technology, in which coal combusts in situ under control and generates combustible gases such as carbon monoxide, hydrogen, and methane through coal pyrolysis and a series of chemical reactions between coal and oxygen, water vapor, and carbon dioxide [1,2,3,4]. This technology can be adopted to recover waste coal resources in old mines and perform in-situ cleaning and conversion of coal resources in deep mines, steeply inclined mines, and mines where traditional technologies are inapplicable, boasting the advantages of remarkable safety, low pollution, and considerable benefits [5,6]. Thanks to these advantages, it has been listed in the *National Energy Technology Revolution Innovation Action Plan (2016–2030)* as a strategic direction of technological innovation for harmless coal mining [7]. One of the key technologies of UGG is the stability of underground gasification and the process of gasification control. The high temperature of 1000–1200 °C in UCG will cause thermal expansion of the rock, resulting in changes in the rock’s physical properties with the variations of temperature [8,9,10,11]. The combined effect of the structural stress associated with the gasification chamber formation and the thermal stress induced by high temperature will damage the rock. When the damage reaches a certain level, the rock will experience the expansion of original fractures and the generation of new fractures, eventually leading to rock fracturing [12,13,14,15,16]. Furthermore, it may cause roof caving, excessive movement, fracturing damage, and surface subsidence of rock strata above the gasification chamber at worst. Meanwhile, the resultant fractures in the overlying strata will cause surface water to rush into the gasification chamber, forcing gas in the gasification chamber to leak or overflow the surface to pollute the environment. As a result, the gasification chamber itself fails to work normally, and even suffers from production stoppage [17]. Since the expansion of fractures in the roof surrounding rock of the UCG gasification chamber is closely related to the thermophysical and mechanical properties of rock strata at high temperature during the gasification process, it is necessary to study the temperature-stress coupling process and the surrounding rock failure law during UCG.

Scholars at home and abroad have conducted extensive research on high-temperature rocks and reaped substantial fruits. For example, Liu and Xu [18] studied the mechanical properties of granite and sandstone at 600 °C after being cooled by water. Tian et al. [19] investigated the basic mechanical properties of rocks at different temperatures (20–600 °C), and explored how their elastic modulus, uniaxial compression strength, and Poisson’s ratio change with temperature. Kong et al. [20] analyzed the influence of high temperatures on rock strength and deformation characteristics. Sun et al. [21] researched the strength and deformation characteristics of sandstone after high-temperature treatment. Lu et al. [22] probed into the variation law of the tensile strength of sandstone at 600 °C under low confining pressure. Kong et al. [23] studied the uniaxial compression strength and acoustic emission law of rocks under high-temperature conditions. Li et al. [24], taking mudstone samples as the research object, found the influence law of temperature on the damage of coal and rock mass by examining the changes in the ultrasonic velocity and porosity of coal and rock mass after the high-temperature treatment. Sirdesai et al. [25] conducted an experiment on the mechanical properties of three kinds of rocks after high-temperature treatment; based on the experimental results, they comparatively analyzed the change laws of the peak stress, peak strain, and elastic modulus of the three types of rocks with temperature and investigated the relationships between peak stress, peak strain, and longitudinal wave velocity. Feng et al. [26] studied the changes in rock mechanical properties and structural effects under the action of temperature and discussed the correlation between changes in rock mechanical properties and structural crystallographic properties under the action of high temperature. Tian et al. [27] investigated the rock strength, rather than rock physical properties, after high-temperature treatment. Yin et al. [28] carried out an experimental study on the effect of high temperature on the rock strength and deformation characteristics. To sum up, studies on high-temperature sandstone and the thermophysical and mechanical properties of rocks after high-temperature treatment remain insufficient. In those few existing studies on sandstone, the heating temperature was controlled in the range of 0–600 °C, which does not conform to the temperature (i.e., around 1000 °C) of sandstone in UGG. Wang et al. [29] studied the mechanical characteristics and crack evolution of granite using discrete element method and Voronoi modeling method.

According to the previous research results, this study takes the Ordos Basin UCG project as the background and collects the roof sandstone samples of the Taiyuan Formation-Shanxi Formation coal seams in the Ordos Basin. With these samples as research objects, high-temperature thermophysical property tests and mechanical experiments under different temperature gradients are systematically conducted. Based on the test results, the sandstone strength and deformation characteristics at different high temperatures are analyzed, and the failure law of roof sandstone after high-temperature treatment is discussed. This study can serve as a foundation for the selection of numerical calculation parameters and engineering application practice of UCG in the Ordos Basin, and boasts guiding significance for a reasonable selection of engineering addresses and safety assessments of UCG.

## 2. Experimental Materials and Methods

### 2.1. Experimental Materials

**Figure 1:**
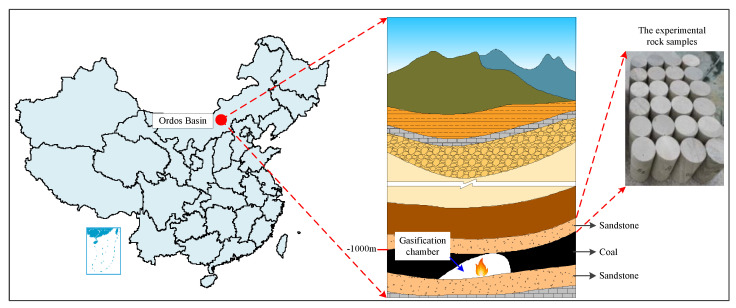
Schematic diagram of the sampling site for sandstone samples [30].

The sampling position of experimental rock samples is illustrated in Figure 1 [30], and they are processed into standard specimens, which are divided into six groups (18 sandstone samples in total) according to different experimental conditions. The specific parameters are shown in Table 1 [30] below.

### 2.2. Experimental Equipment

The MXQ1700 box-type atmosphere oven (produced by Shanghai Weihang Mechanical Equipment Co., Ltd. in China) that features high-temperature control accuracy and low punching temperature is used to heat the samples, and its maximum applied temperature can reach 1700 °C. The STA 449 F5 synchronous thermal analyzer (produced by NETZSCH Instrument Manufacturing Co., Ltd. in Selb, Germany), the Hot Disk TPS2500S thermal constant analyzer (produced by Kegenus Sweden Co., Ltd. in Shanghai, China), and the NETZSCH TMA402F1 thermal mechanical analyzer (produced by NETZSCH Instrument Manufacturing Co., Ltd. in Selb, Germany) were adopted to measure the specific heat capacities, thermal conductivities, and thermal expansion coefficients of rock samples, respectively. The microstructures of the samples were determined using a TESCAN VEGA 3 XMU scanning electron microscope (SEM) system (produced by TESCAN Co., Ltd. in Brno, Czech Republic). The uniaxial compression test of sandstone is carried out by a microcomputer controlled electro-hydraulic servo universal testing machine.

### 2.3. Experimental Procedure

**Figure 2:**
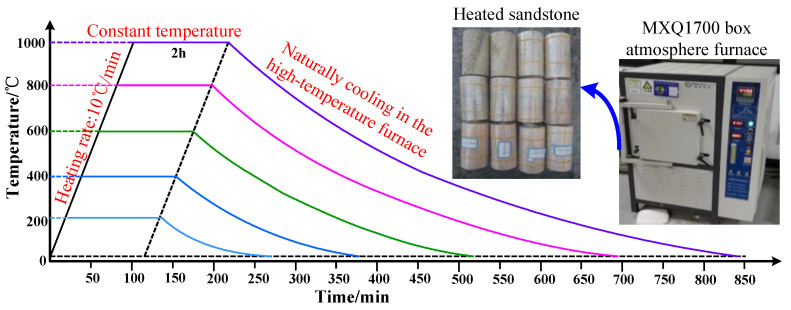
Heating curves of sandstone samples [30].

First, the samples were put into the atmosphere oven with the mouth sealed, and then the oven door was closed. Next, the heating rates were set to 10 °C/min and the heating temperatures were set according to Table 1 [30], with three samples in each temperature gradient. The heating treatment under each temperature gradient lasted for 2 h. After being heated, the samples were not taken out immediately until the oven had naturally cooled down to about 50 °C (Figure 2 [30]). Soon after the samples were removed from the oven, their heights, volumes, and masses were measured. Following this, they were subjected to a uniaxial compression test where displacement control and a loading rate of 0.004 mm/s were adopted. Finally, thermophysical properties such as specific heat capacity, thermal conductivity, and thermal expansion coefficient of these sandstone samples were tested, and their microstructures at different temperatures were observed.

## 3. Analysis of Experimental Results

### 3.1. Influence of High Temperature on Thermophysical Properties of Sandstone

**Figure 3:**
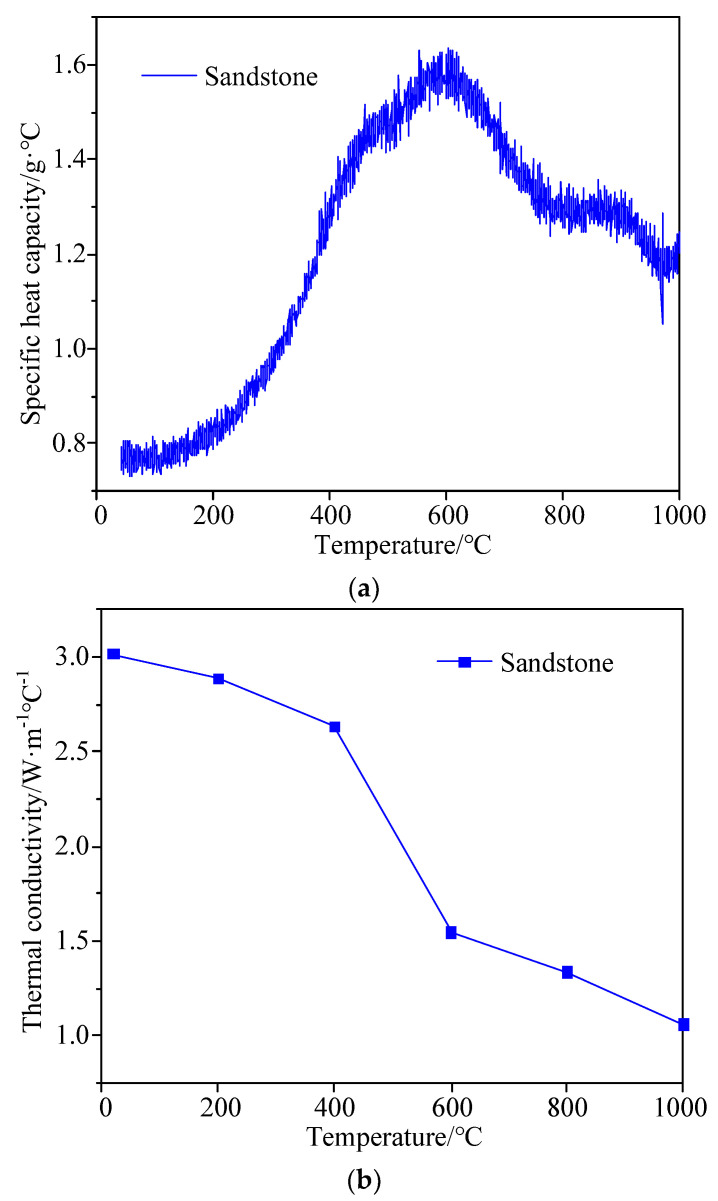

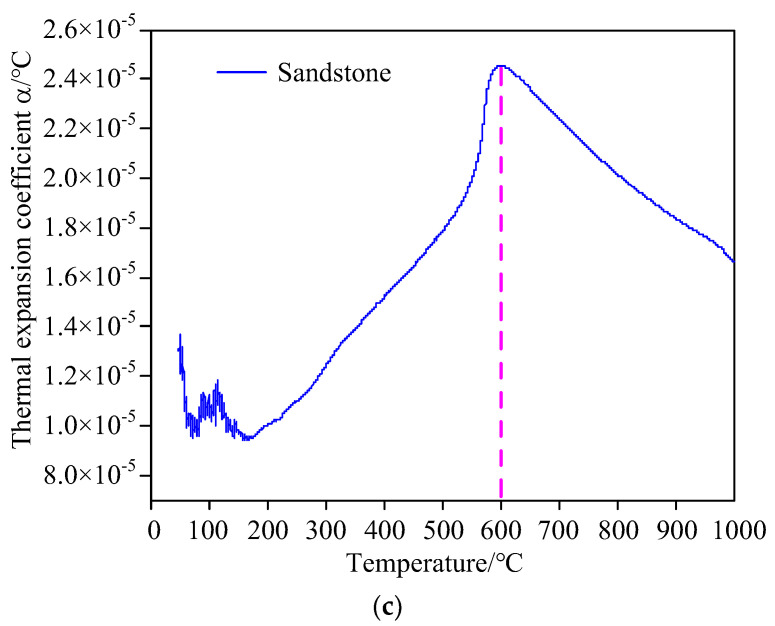
Thermophysical properties of sandstone samples before and after heating. (**a**) Variation curve of specific heat capacity of sandstone samples with temperature; (**b**) Variation curve of thermal conductivity of sandstone samples with temperature; (**c**) Variation curve of thermal expansion coefficient of sandstone samples with temperature.

To study the change in thermophysical properties of sandstone before and after heating, the average values of specific heat capacity, thermal conductivity, and the thermal expansion coefficient of three groups of samples at the same temperature were calculated. Figure 3a shows the variation curve of the specific heat capacity of sandstone samples with temperature. As can be seen in the figure, the specific heat capacity changes significantly after heating, i.e., it first jumps and then declines gradually as the temperature rises. The endothermic peaks, which are observed at around 100 °C and 600 °C, represent the evaporation of free water and the transformation of the quartz phase, respectively. Indeed, the threshold temperature at which the sandstone-specific heat capacity changes significantly is around 600 °C. Figure 3b depicts the variation curve of the thermal conductivity of sandstone samples with temperature. As a whole, the thermal conductivity falls gradually with the increase in temperature. More specifically, the variation of thermal conductivity undergoes three stages: a slow decrease between 25 °C and 400 °C, a rapid decrease between 400 °C and 600 °C and a slow decrease between 600 °C and 1000 °C, among which the rapid decrease in the second stage may be related to the internal composition variation and micro-crack generation in the sandstone samples. Figure 3c presents the variation curve of the thermal expansion coefficient of sandstone samples with temperature. As the temperature rises, two inflection points (100 °C and 600 °C) occur on the curve. At 100 °C, the thermal expansion coefficient is the smallest which is obviously concerned with the evaporation of free water. At 600 °C, the thermal expansion coefficient peaks probably for two reasons: (1) the crystallization of mineral components and the loss of structural water, and (2) the generation of macroscopic cracks inside the sample, i.e., the occurrence of thermal damage, as evidenced by the sharp growth of the thermal expansion coefficient before it reaches the peak.

### 3.2. Analysis of Uniaxial Compression Stress-Strain Curves of High-Temperature Sandstone

**Figure 4:**
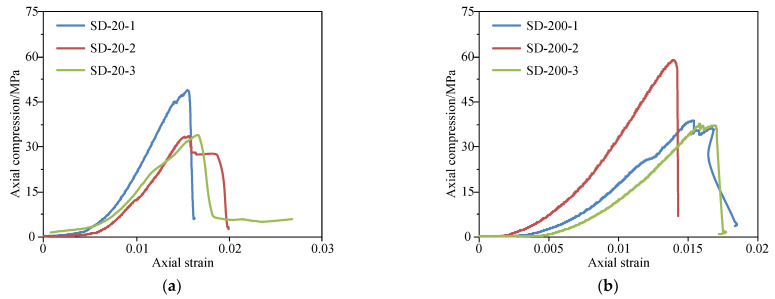

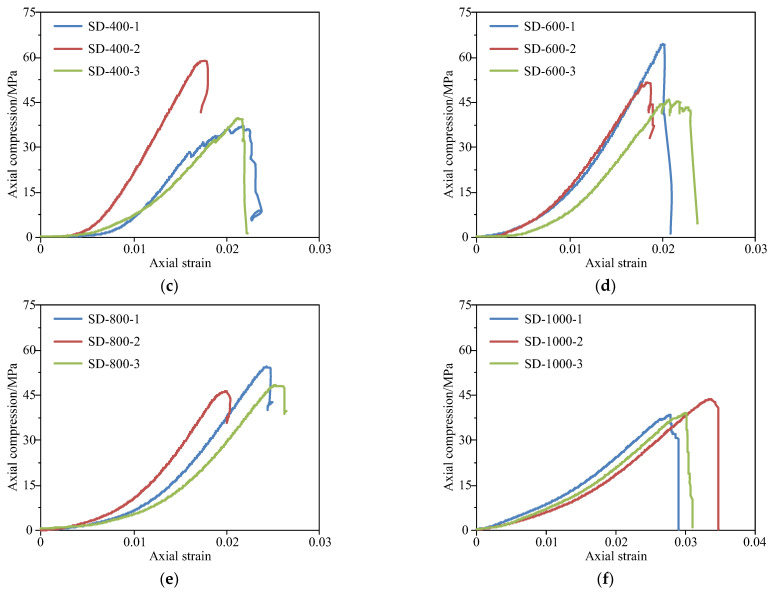
Complete compression stress-strain curves of sandstone under different heating temperatures. (**a**) RT; (**b**) 200 °C; (**c**) 400 °C; (**d**) 600 °C; (**e**) 800 °C; (**f**) 1000 °C.

Figure 4 shows the complete uniaxial compression stress-strain curves of sandstone under different heating temperatures. As presented in Figure 4, the changes in the complete stress-strain curves of sandstone under uniaxial compression after different high temperatures go through roughly four stages: compaction, elasticity, yielding, and failure. In the compaction stage, the curve is concave and the deformation develops quickly with the increase of compression stress, due to the rapid closure of microcracks in the rock under the action of loading. Indeed, it can also be seen that the higher the temperature, the longer the compaction stage, which indicates that the generated microcracks grow with the rise of the temperature. Then comes the elasticity stage, in which the compression stress and strain share a proportional relationship. In this stage, the curve is basically a straight line whose slope is the average tangent elastic modulus of the rock. Next, it enters the plastic deformation stage, in which the compression increases little while the strain rises dramatically. Moreover, the higher the temperature, the more obvious the plastic deformation is. The last stage is the failure stage, where the axial compression stress reaches the peak, and the sandstone is damaged, but it still possesses certain bearing capacity.

### 3.3. Variation Law of Peak Strength with Temperature

**Figure 5:**
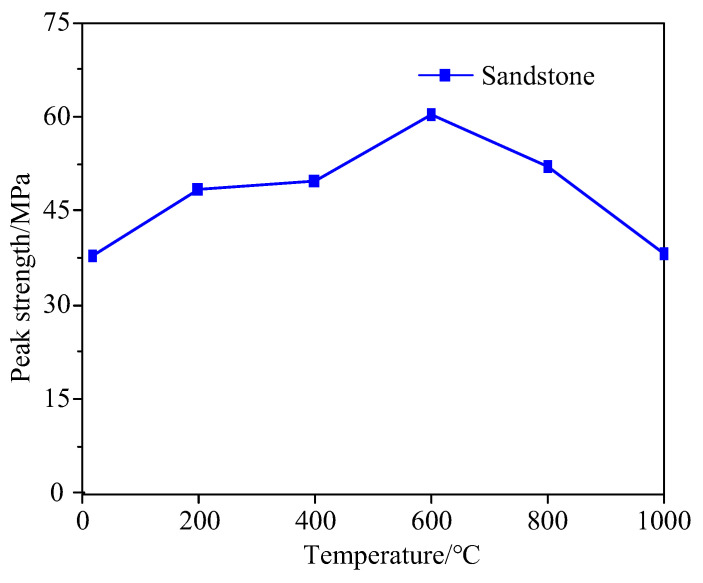
Variation curve of sandstone peak strength with temperature.

Figure 5 depicts the relationship between the peak strength of sandstone and temperature. As revealed by Figure 5, the peak strength of sandstone, as a whole, rises first and then falls with the increase in temperature despite the discreteness of peak strength at the same temperature. Before 600 °C, the peak strength increases as the temperature rises, with the average value increasing by 44% from 36.89 MPa to 53.14 MPa. This is because the thermal stress generated at the high temperature allows the internal space of the rock to accommodate deformation and prevent crack expansion. On top of this, the thermal expansion of mineral particles inside the rock may also force the preexisting cracks in the sandstone to gradually close, thus decreasing the number of cracks in the sandstone, improving the degree of compaction, and enhancing the peak strength. At 600–1000 °C, the peak strength plunges by 30%, mainly because the thermal stress of the internal structure of the sandstone reaches or exceeds the tolerable strength of the material after high-temperature treatment. Under such circumstances, microcracks occur in the rock, weakening the bearing capacity and anti-deformation ability of sandstone samples. Indeed, as the temperature continues to rise, the influence of thermal stress becomes more apparent, which will induce the appearance of more microcracks inside the sandstone. At the same time, the preexisting cracks expand, extend and connect under the action of thermal stress, which reduces the peak strength of the sandstone.

### 3.4. Variation Law of Peak Strain with Temperature

**Figure 6:**
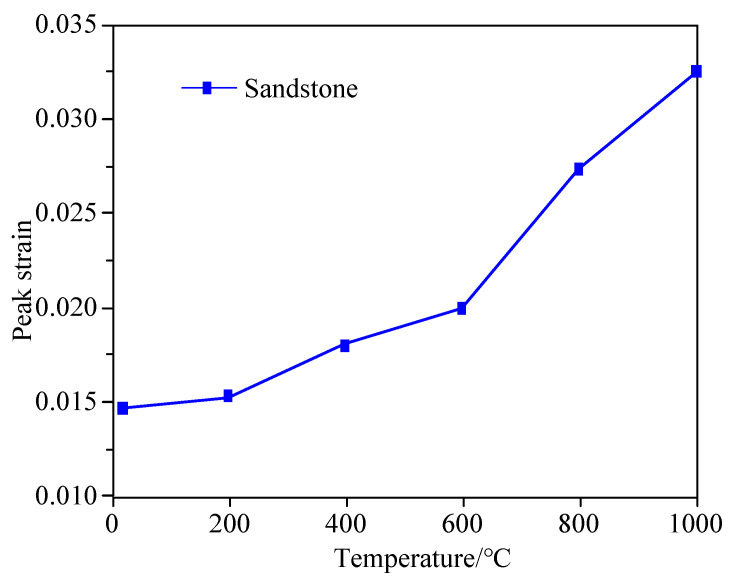
Variation curve of sandstone peak strain with temperature.

The variation of peak strain of sandstone with temperature after high-temperature treatment is presented in Figure 6. As illustrated in Figure 6, the increase in the temperature accelerates the thermal motion of molecules in the sandstone and weakens their cohesion as the ductility of the sandstone is improved due to the high temperature, which makes it easier for the grain surface of the sandstone to slip. The higher the temperature, the more likely it is for microcracks to emerge in the sandstone, so the peak strain of the sandstone increases as the temperature rises. Within 200 °C, the peak strain witnesses no significant change. When the temperature increases from 200 °C to 600 °C, the peak strain rises gently by 30.0% with the increase in temperature. When the temperature exceeds 600 °C, the peak strain soars by 59.9% as the temperature increases.

### 3.5. Variation Law of Modulus of Elasticity with Temperature

**Figure 7:**
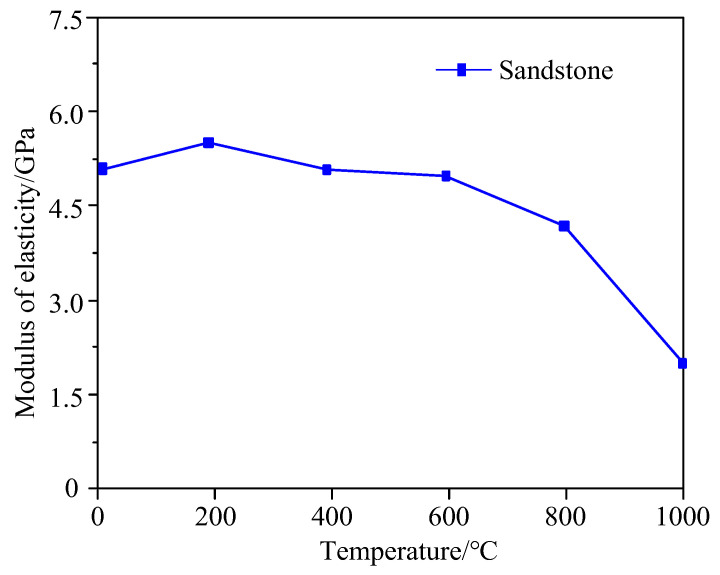
Variation curve of modulus of elasticity of sandstone with temperature.

To explore the relationship between the modulus of elasticity and temperature, the slope of the line connecting 0.03 times the peak stress and 0.7 times the peak stress is taken as the modulus of elasticity. Figure 7 shows the variation curve of the modulus of elasticity of sandstone with temperature.

As demonstrated in Figure 7, the following phenomena can be observed: The modulus of elasticity decreases with the increase of temperature as a whole. When the temperature rises from RT to 200 °C, the modulus of elasticity grows from 4.59 GPa to 4.97 GPa by 8.3%. When the temperature ranges from 200 °C to 400 °C, it decreases from 4.97 GPa to 4.57 GPa by 8.1%. In the range of 400–600 °C, it declines from 4.57 GPa to 4.49 GPa by 4.8% with the increase in temperature. When the temperature is greater than 600 °C, it plunges from 4.49 GPa to 1.78 GPa (equal to the value at 100 °C) by 60.4%. In short, the modulus of elasticity of sandstone reaches the maximum at 200 °C, declines slowly in the range of 200–600 °C, and plunges when the temperature exceeds 600 °C. Furthermore, it is also concluded that the higher the temperature, the more noticeable the modulus of elasticity changes.

### 3.6. Analysis of the Microstructure of Sandstone after High-Temperature Treatment

**Figure 8:**
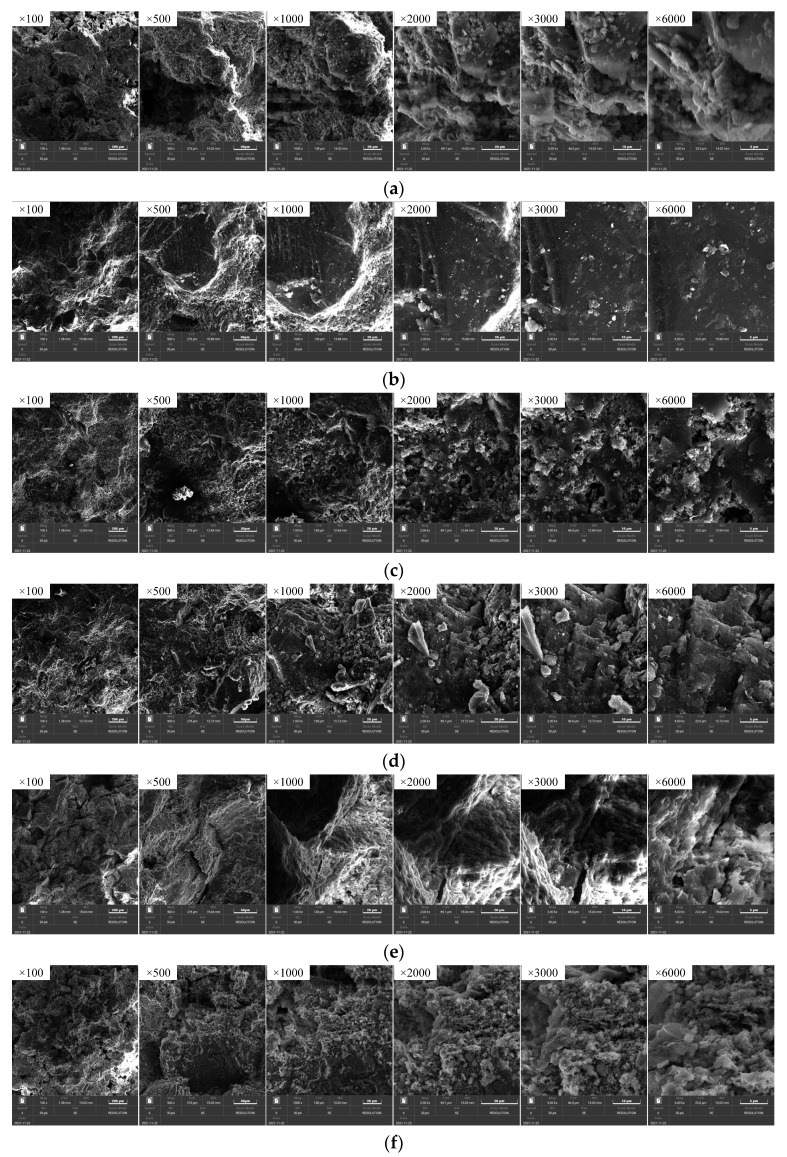
SEM images of sandstone after different high temperatures. (**a**) RT; (**b**) 200 °C; (**c**) 400 °C; (**d**) 600 °C; (**e**) 800 °C; (**f**) 1000 °C.

The SEM images of sandstone after different high-temperature treatments are exhibited in Figure 8. As shown in Figure 8a, the particles of sandstone, with many pores and cracks inside, are not tightly bound at RT, but the sample is quite integrated with a smooth surface and high strength. As illustrated in Figure 8b, after the sandstone is heated at 200 °C, its internal cracks stop expanding further as a result of the thermal stress. Meanwhile, the thermal expansion of mineral particles may also cause the preexisting cracks in the sandstone to gradually close, reducing the number of rock cracks and improving the degree of compaction. Therefore, sandstone boasts a higher rock strength and resistance to deformation. According to Figure 8c–e, the ablation traces of the sandstone gradually become apparent as the temperature rises, which is marked by the rough particle surfaces and the increase of powder particles. Moreover, small intergranular cracks appear in some areas, which are cracks caused by the volumetric expansion and contraction before and after quartz phase transformation. As revealed in Figure 8f, when the temperature reaches 1000 °C, melting marks are observed on the sandstone surfaces, the boundaries of minerals are blurred, and the edges and corners of the particles vanish. This is due to the fact that, as the thermal stress of the structure exceeds the ultimate strength of the material, microcracks are generated inside the sandstone samples, causing the mechanical properties to deteriorate.

## 4. Discussion

In terms of the deterioration of mechanical properties, temperature exerts a great influence on the mechanical properties and crack structure of sandstone, which further plays a decisive role in the stability of the roof of the the UCG gasification chamber. Therefore, studying the mechanical properties of sandstone after high-temperature treatment is of positive significance for UCG.

Based on the above analysis of experimental results, the main mechanisms of changes in the mechanical properties of sandstone after high-temperature treatment include water loss and structural damage induced by the thermal reaction. When the sandstone samples are heated to 200 °C, the sandstone density drops slightly under the influence of thermal expansion and free water evaporation, and overall rock mechanical parameters are improved due to the compaction of pores. In the stage of 200–400 °C, some minerals in the sandstone are ablated, resulting in a continuous decrease in density. However, under the combined action of thermal expansion and ablation, the mechanical parameters of the sandstone change little. When the sandstone samples are heated to 400–600 °C, the internal constitutional water of the sandstone is lost, and defects increase. Moreover, organic matter such as carbon and sulfur will undergo thermal decomposition and other thermochemical reactions, further reducing the rock density. At the same time, quartz will be transformed from the α phase to the β phase when the temperature approaches 600 °C, during which the volume expands slightly and the impurities in the quartz crystal are purified. Such a transformation would result in significant changes in the physical and mechanical properties of the sandstone as a result of its high content of quartz, for example, its compressive strength and viscosity between the matrices will peak. In the stage of 600–800 °C, many minerals in the sandstone begin to experience melting and phase transition. For example, the chemical bonds such as Al-O, K-O, Na-O, and Ca-O in the minerals break, and some minerals in the sample melt, which leads to the appearance of many microscopic defects in the rock, and even macroscopic cracks. Consequently, rock density drops continuously and the rock strength plunges. When the sandstone samples are heated to 800–1000 °C, the thermal reaction of clay minerals in them is enhanced and the reorganization of minerals brings about the disappearance of edges and corners of mineral particles, notable changes in the morphology of pores and cracks, as well as a rapid fall of the mechanical strength of sandstone.

The research results suggest that temperature has a significant impact on the mechanical properties of sandstone. However, the evolution law of sandstone mechanical properties with temperature varies, so future research will focus on the comparison of sandstone mechanical properties after different high-temperature treatments. Moreover, analyzing the micro-mechanism of mechanical property evolution with temperature is also the fundamental way to explore the law of permeability evolution and uncover its essential causes.

## 5. Conclusions and Suggestions

1. Temperature has an enormous influence on the thermophysical properties of sandstone. Specifically, the specific heat capacity and thermal expansion coefficient first rise and then fall as the temperature increases, which falls into three stages: slow increase in the range of RT-200 °C, a sharp increase in the range of 200–600 °C, and a plunge in the range of 600–1000 °C. In contrast, the thermal conductivity drops gradually with the increase in temperature. The thermophysical properties of sandstone do not change remarkably in the range of RT-200 °C, but they witness significant changes in the range of 200–1000 °C. Indeed, the higher the temperature, the more notable the changes.
2. The mechanical properties of sandstone after high-temperature treatment are closely related to temperature, which is reflected in the following changes: the peak strength of sandstone increases when the temperature rises from RT to 600 °C, but decreases when the temperature rises from 600 °C to 1000 °C. As the temperature rises from RT to 1000 °C, the peak strength of sandstone grows, while the modulus of elasticity of sandstone drops with a steeper margin of decrease when the temperature exceeds 600 °C.
3. Temperature affects both the strength of sandstone and its microstructure. Under the action of high temperatures above 200 °C, the internal cracks of the sandstone begin to develop. When the temperature is higher than 600 °C, obvious cracks appear in the sandstone crystal. The increase in crack density would cause the loosening of rock samples and gradual decreases in compressive strength and modulus of elasticity. When the temperature reaches 1000 °C, melting marks appear on the sandstone surface and the mineral boundary is blurred. In addition, the compressive strength and modulus of elasticity of the sandstone samples see an abrupt decrease.

## Figures and Tables

**Table 1:** Physical parameters of rock samples [30].

Sample Temperature	Sample No.	Diameter/mm	Height/mm	Mass/g	
				before Heating	after Heating
Room temperature	SD-20-1	49.0	99.5	469.01	469.01
	SD-20-2	49.5	99.2	450.51	450.51
	SD-20-3	50.2	95.9	470.25	470.25
200 °C	SD-200-1	49.7	101.4	453.30	452.15
	SD-200-2	50.4	100.8	454.15	453.08
	SD-200-3	50.3	100.2	455.34	454.32
400 °C	SD-400-1	49.1	96.4	458.51	446.85
	SD-400-2	50.9	97.8	429.58	428.53
	SD-400-3	49.7	95.6	460.95	459.90
600 °C	SD-600-1	50.2	98.3	459.93	456.78
	SD-600-2	50.7	97.2	461.57	458.84
	SD-600-3	49.9	100.7	453.11	451.00
800 °C	SD-800-1	50.0	100.2	457.86	455.20
	SD-800-2	50.7	97.5	449.62	447.03
	SD-800-3	50.2	98.2	451.94	448.10
1000 °C	SD-1000-1	50.7	101.4	455.22	453.87
	SD-1000-2	49.2	99.5	440.60	437.47
	SD-1000-3	49.5	97.7	451.63	449.81

## Data Availability

The data used to support the findings of this study are included within the article.

## References

[1] Sun Q., Geng J.S., Zhao F. (2020). Experiment study of physical and mechanical properties of sandstone after variable thermal cycles. Bull. Eng. Geol. Environ..

[2] Jin P.H., Hu Y.Q., Shao J.X. (2020). Influence of Temperature on the Structure of Pore-Fracture of Sandstone. Rock Mech. Rock Eng..

[3] Meng F.D., Li Y.B., Zhai Y. (2022). Study on the Effect of Sandstone Microscopic Damage and Dynamic Compressive Properties After Heat Treatment. Rock Mech. Rock Eng..

[4] Emirov S.N., Aliverdiev A.A., Zarichnyak Y.P. (2021). Studies of the Effective Thermal Conductivity of Sandstone Under High Pressure and Temperature. Rock Mech. Rock Eng..

[5] Zhang W.Q., Wang Z.Q., Du Y., Zhang S.T. (2022). Effect of high temperature on pore characteristics, yield stress, and deformation property of sandstone. Bull. Eng. Geol. Environ..

[6] Pan X.K., Berto F., Zhou X.P. (2022). Creep damage behaviors of red sandstone subjected to uniaxial compression after high-temperature heat treatment using acoustic emission technology. Fatigue Fract. Eng. Mater. Struct..

[7] Li M., Wang D.M., Shao Z.L. (2020). Experimental study on changes of pore structure and mechanical properties of sandstone after high-temperature treatment using nuclear magnetic resonance. Eng. Geol..

[8] Yuan S.H., Sun Q., Li P.F., Geng J.S. (2022). Fracture properties and dynamic failure of three-point bending of yellow sandstone after subjected to high-temperature conditions. Eng. Fract. Mech..

[9] Zhang R.R., Yi Y., Ma D.D. (2021). Investigation on Damage Characteristic and Constitutive Model of Deep Sandstone under Coupled High Temperature and Impact Loads. Geofluids.

[10] Xiao W.J., Yu G., Li H.T., Zhang D.M. (2021). Thermal cracking characteristics and mechanism of sandstone after high-temperature treatment. Fatigue Fract. Eng. Mater. Struct..

[11] Jing X.D., Sun Q., Jia H.L. (2021). Influence of high-temperature thermal cycles on the pore structure of red sandstone. Bull. Eng. Geol. Environ..

[12] Jiang H.P., Jiang A.N., Zhang F.R. (2021). Experimental investigation on the evolution of damage and seepage characteristics for red sandstone under thermal-mechanical coupling conditions. Environ. Earth Sci..

[13] Huang Y.H., Yang S.Q., Dong J.P. (2020). Experimental study on fracture behaviour of three-flawed sandstone specimens after high-temperature treatments. Fatigue Fract. Eng. Mater. Struct..

[14] Liu G., Chen Y., Du X., Wang S., Fernández-Steeger T.M. (2022). Evolutionary Analysis of Heterogeneous Granite Microcracks Based on Digital Image Processing in Grain-Block Model. Materials.

[15] Lei R.D., Wang Y., Zhang L., Liu B.L. (2019). The evolution of sandstone microstructure and mechanical properties with thermal damage. Energy Sci. Eng..

[16] Daraei A., Zare S. (2018). Effect of Water Content Variations on Critical and Failure Strains of Rock. KSCE J. Civ. Eng..

[17] Fan L.F., Li H., Xi Y. (2022). Evaluation of the effects of three different cooling methods on the dynamic mechanical properties of thermal-treated sandstone. Bull. Eng. Geol. Environ..

[18] Liu S., Xu J.Y. (2015). An experimental study on the physico-mechanical properties of two post-high-temperature rocks. Eng. Geol..

[19] Tian H., Kempka T., Yu S. (2016). Mechanical Properties of Sandstones Exposed to High Temperature. Rock Mech. Rock Eng..

[20] Kong B., Wang E.Y., Li Z.H. (2016). Electromagnetic radiation characteristics and mechanical properties of deformed and fractured sandstone after high temperature treatment. Eng. Geol..

[21] Sun Q., Lu C., Cao L.W. (2016). Thermal properties of sandstone after treatment at high temperature. Int. J. Rock Mech. Min. Sci..

[22] Lu C., Sun Q., Zhang W.Q. (2017). The effect of high temperature on tensile strength of sandstone. Appl. Therm. Eng..

[23] Kong B., Wang E.Y., Li Z.H. (2016). Fracture Mechanical Behavior of Sandstone Subjected to High-Temperature Treatment and Its Acoustic Emission Characteristics Under Uniaxial Compression Conditions. Rock Mech. Rock Eng..

[24] Li M., Mao X.B., Cao L.L. (2016). Effects of Thermal Treatment on the Dynamic Mechanical Properties of Coal Measures Sandstone. Rock Mech. Rock Eng..

[25] Sirdesai N.N., Singh T.N., Ranjith P.G. (2017). Effect of Varied Durations of Thermal Treatment on the Tensile Strength of Red Sandstone. Rock Mech. Rock Eng..

[26] Feng G., Kang Y., Meng T. (2017). The Influence of Temperature on Mode I Fracture Toughness and Fracture Characteristics of Sandstone. Rock Mech. Rock Eng..

[27] Tian H., Kempka T., Xu N.X. (2012). Physical Properties of Sandstones After High Temperature Treatment. Rock Mech. Rock Eng..

[28] Yin T.B., Wang P., Yang J. (2018). Mechanical Behaviors and Damage Constitutive Model of Thermally Treated Sandstone Under Impact Loading. IEEE Access.

[29] Wang S., Chen Y., Xiong M., Du X., Liu G., Fernández-Steeger T.M. (2021). The Mechanism of Fracture and Damage Evolution of Granite in Thermal Environment. Materials.

[30] Ding K., Wang L., Ren B., Li Z., Wang S., Jiang C. (2021). Experimental Study on Relative Permeability Characteristics for CO_2_ in Sandstone under High Temperature and Overburden Pressure. Minerals.

